# Supplementary material for: Examining outdoor play associations in Canadian early learning and child care centres: Cross-sectional insights from the Measuring Early Childhood Outside survey
Source: PLoS One. 2026 Feb 11;21(2):e0331166. doi: 10.1371/journal.pone.0331166 (PMC12893580; doi:10.1371/journal.pone.0331166)
Supplement: S1 Appendix — (PDF) [file pone.0331166.s001.pdf]

S1 Appendix: MECO Survey Development, Testing and Administration

Survey Pilot Testing

The MECO survey was developed with input from a literature review and the advisory committee. A literature review was conducted to find existing survey research that could inform survey development and survey questions previously used to measure children's OP provision. The study was guided by an advisory committee that included individuals from ELCC organizations and academic institutions across Canada. The committee set priorities for the survey, reviewed survey drafts, recommended recruitment strategies, and assisted with interpreting the results. The MECO survey was developed in English and subsequently translated into French. The English and French versions of the survey were hosted on REDCap (Harris et al., 2009), a secure, password-protected data collection platform hosted through the [redacted for review]. To ensure clarity and usability, the survey was pilot-tested with 9 ELCC providers (5 English-speaking and 4 French-speaking) through cognitive interviews. These interviews helped evaluate the content and comprehension of survey questions. Based on feedback from these sessions, the survey was iteratively revised. The final survey included eligibility screening questions and questions relating to the ELCC centre characteristics (e.g., auspice, staffing, size), program type, characteristics of the children attending the program, days and hours spent in OP (in summer and winter), factors influencing OP time (e.g. licensing regulations, weather, etc.), characteristics of the outdoor space (e.g., size, access, loose and fixed parts), permitted outdoor risky play activities, other outdoor locations, provision of all-weather gear, and staff training and experience. Respondents were also invited to upload photos of their outdoor space, and share any other relevant information. The administered version of the MECO survey is provided in Supplementary Material 1.

Survey Distribution and Promotion

The [redacted for review] provided approval for the research (#H22-00210). To distribute the survey, a comprehensive list of eligible ELCC programs across Canada was compiled from public databases, government contacts, and regional organizations. Where direct email contact information could not be obtained, outreach was done through trusted provincial or regional organizations. The survey was distributed via email to all provinces and territories, except Alberta and Ontario. In these two provinces, alternative strategies were used (e.g., social media promotion, collaboration with local advocacy groups, and posting in community newsletters) due to the lack of publicly available or easily reachable contacts. Additionally, no outreach was done to ELCCs in the Northwest Territories (NWT) as we were unable to obtain a research license to become eligible to collect data in the NWT. Social media promotion was initially employed but halted in August 2023 due to an influx of automated (i.e., ‘bot’) responses. Ongoing promotion included reminder emails sent to regional, provincial, territorial, and national organizations, and through newsletters such as the OP Canada Newsletter and e-blasts from the Canadian Child Care Federation. The survey was also promoted at the Breath of Fresh Air Conference in September 2023.

Survey Administration

The MECO survey was conducted between June and October 2023 in both English and French. We requested that the survey be completed by only one respondent for each ELCC centre and required that the respondent was the individual most responsible for the day-to-day operations of the centre. If respondents were responsible for the operation of multiple centres in separate physical addresses, they were instructed to complete a separate survey for each centre.

Respondents who completed the survey were incentivized with a $25 gift card. To ensure data quality, enhanced security features were added to the survey (reCAPTCHA technology), email authentication, and ‘honey pot’ style verification questions tailored to ELCC-specific knowledge. Further, the research team validated responses by screening for automated bot submissions and duplicate responses. Examples of screening methods to eliminate automatic bot responses include examining overall survey response time (<3 minutes was determined as not possible for verified responses), reviewing responses to ‘honey pot’ questions, and examining open-text and photo submissions for artificially generated or stock responses. After the survey closed, verified responses were de-identified, assigned unique IDs, and exported from the REDCap platform for analysis.

Measuring Early Childhood Outside Survey

Landscapes for Outdoor Play in Canadian Early Learning and Childcare Centres: a mixed methods study

You are invited to participate in the Measuring Early Childhood Outside (MECO) survey as part of the Landscapes for Outdoor Play in Canadian Early Learning and Childcare Centres study. The purpose of this survey is to understand children's outdoor play practices and the associated supports and challenges that influence outdoor play in child care centres across Canada.

The principal investigator of this study is Dr. Mariana Brussoni from the BC Children's Hospital Research Institute and the Human Early Learning Partnership at the University of British Columbia. Ethics certification for this research was received from the University of British Columbia and the Children's and Women's Health Centre of British Columbia Research Ethics Board (study number: H22-00210). This project is generously supported by the Lawson Foundation, the Lyle S. Hallman Foundation, and the Muttart Foundation.

This survey is being sent to all licensed child care centres providing full-day care for children not yet in Kindergarten in Canada. Participation in this survey is voluntary and you may stop the survey at any time without any penalty. Please note that original survey data is stored in REDCap, and then will be exported and saved in a password-protected folder in a secured network at the BC Children's Hospital Research Institute. No information revealing your identity will be disclosed or published. All responses will be deidentified and only grouped results will be reported. Deidentified data may be made available to other parties through an open-access data repository. Please contact the research team for further questions on confidentiality. There are no anticipated risks of participating in this survey.

This survey takes about 15-20 minutes to complete. If you require more than one session to complete it, you can return to the survey without having to start over, as long as you use the same computer or smartphone to complete it. The first 1,000 participants will be eligible to receive a $25 gift card upon full completion of the survey. Verification of responses may be requested by the research team prior to receiving your gift card. Limit of one gift card per person.

You are eligible to complete this survey if:
- You are the individual most responsible for the overall daily operation of a licensed full day, group care, child care centre in Canada;
- You are 19 years of age or older; and
- Can read and understand English or French.

If this description does not fit you, please pass this survey to the person at your child care centre who best fits this description.

Further information about this survey can be found at: https://playoutsideubc.ca/measuring-early-childhood-outside/

If you have any questions or concerns about what we are asking of you, please contact Dr. Mariana Brussoni at mbrussoni@bcchr.ca or 604-875-3712, or Rachel Ramsden, Research Coordinator at rramsden@bcchr.ca or 604-875-2000 ext 2433.

If you have any concerns or complaints about your rights as a research participant and/or your experiences while participating in this study, contact the Research Participant Complaint Line in the UBC Office of Research Ethics at 604-822-8598 or if long distance e-mail RSIL@ors.ubc.ca or call toll free 1-877-822-8598. Please reference the study number H22-00210 when contacting the Complaint Line so the staff can better assist you.

By submitting the completed survey, you are consenting to participate in this research.

Which types of regulated/licensed child care do you offer?
[select all that apply]

- ☐ Licensed full-day child care centre
- ☐ Part-day preschool or nursery school
- ☐ Part-day school-age child care
- ☐ Home-based (family) child care

Are you the person most responsible for the day-to-day operation of this child care centre (e.g. director, administrator, supervisor, etc.)? [select one]	◯ Yes<br>◯ No
Is this centre a part of a multi-site child care organization? [select one]	◯ Yes<br>◯ No
Is this centre a part of a multi-service agency that also offers other social/family services? [select one]	◯ Yes<br>◯ No
Are you responding on behalf of more than one child care centre, or more than one (i.e. for an organization with multiple child care sites)? [select one]<br><br>Note: If you have multiple licences for programs in the same location, please treat them as one centre.	◯ No, just for one centre<br>◯ Yes, for multiple centres in our organization

We would like a separate response for each child care centre in your organization. We would appreciate it if you could forward this survey to the person most responsible for the day-to-day operations at each individual centre that your organization operates. Here is the shareable link for your convenience: https://rc.bcchr.ca/redcap/surveys/?s=7TPN4KHLTDYCEDNN

If you would like to respond on behalf of multiple child care centres, please complete this survey for one centre at a time. At the end of each survey, you will be able to complete the survey again for a new centre. Please continue if you would like to complete this survey for one centre.

If you have any questions, please email outdoorplay@bcchr.ca.

What are your day-to-day roles at the centre? [select all that apply]	☐ Owner<br>☐ Director, manager, supervisor, or lead educator not working directly with children<br>☐ Director, manager, supervisor, or lead educator working directly with children<br>☐ Educator working directly with children<br>☐ Bookkeeping, cooking, or janitorial<br>☐ Other
Other (please specify)	__________________________________
What are the first three characters of the postal code of the physical address of this centre? [number or text entry]	__________________________________
How is this child care centre operated? [select all that apply]	☐ As a not-for-profit<br>☐ By a municipality, a public school authority/board, or provincial or federal agency<br>☐ By an Indigenous-led not-for-profit<br>☐ By an Indigenous government<br>☐ As a private, for-profit business (incorporated)<br>☐ As a private, for-profit business (sole proprietor or partnership)<br>☐ Other (please specify)
Other (please specify)	__________________________________

Which age groups, as defined by your provincial/territorial regulations, are you licensed to care for in your full-day child care program(s) at this centre?
[select all that apply]

- ☐ Infant and/or toddler-aged children
- ☐ Preschool-aged children

Infant & Toddler-Aged Children Responses:

Please respond to the following questions regarding the licensed, full-day child care program that serves infant and toddler-aged children.

How many infant and/or toddler spaces is this licensed, full-day program licensed for (ie. licensed capacity)?
[number entry] ________________________________

How many infant and/or toddlers are enrolled full-time, full-day in this program?
[number entry] ________________________________

How many infant and/or toddlers are enrolled part-time or part-day in this program?
[number entry] ________________________________

If this program has a licensed capacity that requires further description, please let us know here.
[text entry] ____

How many infant and/or toddlers in this program have a diagnosed disability?
[number entry] ________________________________

How many infant and/or toddlers in this program have a primary language that is not English or French?
[number entry] ________________________________

How many infant and/or toddler families in this program identify as Indigenous (First Nations, Métis, or Inuit)?
[number entry] ________________________________

In a typical week in each season, how many days are infants and toddlers taken outdoors?

	0 days	1 day	2 days	3 days	4 days	5 or more days
Fall	○	○	○	○	○	○
Winter	○	○	○	○	○	○
Spring	○	○	○	○	○	○
Summer	○	○	○	○	○	○

On a typical day in each season, approximately how many hours does each infant/toddler spend outdoors?

	None	1 hour	1-2 hours	2-3 hours	3-4 hours	5 or more hours

Fall	◯	◯	◯	◯	◯	◯
Winter	◯	◯	◯	◯	◯	◯
Spring	◯	◯	◯	◯	◯	◯
Summer	◯	◯	◯	◯	◯	◯

On a typical day, how is time for outdoor play usually allocated within this infant and toddler-aged program? [select one]

- ◯ According to a fixed schedule
- ◯ Spontaneously by the educators
- ◯ Mix of scheduled and spontaneous

On a typical day, what is the usual group composition for outdoor play in this infant and toddler-aged program? [select one]

- ◯ Children go outside in one small group
- ◯ Children go outside in two small groups
- ◯ Children go outside in one large group
- ◯ Children go outside in both small groups and as a large group
- ◯ Other (please indicate)

Other (please indicate)

____

How often are children able to decide when they want to go outside in this infant and toddler-aged program? [select one]

- ◯ Often/always
- ◯ Sometimes/occasionally
- ◯ Rarely/never

(Examples - Often/ always: If a child does not want to go outside, they do not have to; or if a child wishes to stay outside longer, they are allowed to. Rarely/ never: Child does not determine when they go outside - this may be set by staff ratios/availability or the schedule for the day.)

How often are infant and toddler-aged children taken outdoors at the following times of the day in the summer months (June - September)?

	Never	Rarely	Occasionally	Often	Always
Early morning (before 10:00am)	◯	◯	◯	◯	◯
Late morning (10:00am - 12:00pm)	◯	◯	◯	◯	◯
Afternoon (12:00pm - 2:00pm)	◯	◯	◯	◯	◯
Late afternoon (2:00pm - 4:00pm)	◯	◯	◯	◯	◯
Evening (after 4:00pm)	◯	◯	◯	◯	◯

How often are infant and toddler-aged children taken outdoors at the following times of the day in the winter months (December - March)?

	Never	Rarely	Occasionally	Often	Always
Early morning (before 10:00am)	◯	◯	◯	◯	◯
Late morning (10:00am - 12:00pm)	◯	◯	◯	◯	◯
Afternoon (12:00pm - 2:00pm)	◯	◯	◯	◯	◯
Late afternoon (2:00pm - 4:00pm)	◯	◯	◯	◯	◯

Evening (after 4:00pm)	◯	◯	◯	◯	◯

In your opinion, how important are the following factors in determining how much time infant and toddler-aged children spend outdoors in this program?

	Not Important	Neutral	Important	Very Important	Not Applicable
Educator professional development and training to support outdoor play	◯	◯	◯	◯	◯
Educators are interested in being outdoors and supporting outdoor play	◯	◯	◯	◯	◯
Staffing resources and availability of staff to spend time outdoors	◯	◯	◯	◯	◯
Families support their children playing outside	◯	◯	◯	◯	◯
Size and quality of outdoor play area	◯	◯	◯	◯	◯
Availability of the outdoor play area (e.g. must share space with other programs)	◯	◯	◯	◯	◯
Weather patterns and/or availability of weather-appropriate attire for children and staff	◯	◯	◯	◯	◯
Licensing regulations and requirements	◯	◯	◯	◯	◯
Support for children who require extra support or may exhibit challenging behaviours	◯	◯	◯	◯	◯

Are there other factors that influence how much time children spend outdoors in this program (please indicate)? ______________________________

Do children in this infant/toddler program access the licensed outdoor play area directly from the licensed indoor area?
[select one]

◯ Yes
◯ No

Does this infant/toddler program currently receive any amendments, variances or exemptions to licensing requirements to meet licensing regulations for outdoor play?
[select one]

◯ Yes
◯ No

Yes (please describe) ______________________________

Is there an outdoor space that is off-site that is used to meet licensing requirements for this infant/toddler program?<br>[select one]	◯ Yes<br>◯ No
What type of off-site outdoor space is used to meet licensing requirements?	__________________________________
On average, how many minutes does it take infants and toddlers in this program to walk from the indoor space to the off-site outdoor space that is used to meet licensing requirements?<br>[number entry]	__________________________________
Approximately how big is this infant and toddler-aged program's outdoor play area?<br>[select one]	◯ Less than required by licensing regulations (sq. m/child)<br>◯ Exactly what is required by licensing regulations (sq. m/child)<br>◯ Slightly larger than what is required by licensing regulations (sq. m/child)<br>◯ Much larger than what is required by licensing regulations (sq. m/child)
If you are able, please provide the approximate size (square metres) of the outdoor play area that is used by this infant and toddler-aged program.<br>[number entry]	__________________________________<br>(Square metres (approximate))
On the sunniest days of the year, what is the proportion of your outdoor play area for infants and toddler-aged children that receives shade coverage by structures or trees?<br>[select one]	◯ No shade<br>◯ 25% to 50% is shaded<br>◯ 50% to 75% is shaded<br>◯ More than 75% is shaded
Does the infant/toddler outdoor play area have a surrounding fence?<br>[select one]	◯ Yes<br>◯ No
What types of surfacing does the infant/toddler outdoor play area have?<br>[select all that apply]	☐ Artificial grass<br>☐ Bark mulch or engineered wood fibre<br>☐ Grass<br>☐ Concrete<br>☐ Paving stones or tiles<br>☐ Rubber<br>☐ Sand<br>☐ Pea gravel<br>☐ Decking<br>☐ Pebbles<br>☐ Asphalt<br>☐ Dirt<br>☐ Mud<br>☐ Other (please specify)
Other (please specify)	__________________________________

Which of following types of portable equipment and materials are available for infant and toddler-aged children to regularly use outdoors?<br>[select all that apply]	☐ Jumping items (e.g. jump ropes, jumping balls)<br>☐ Push-pull items (e.g. wagons, wheelbarrows, big dump trucks)<br>☐ Ride on items (e.g. tricycles, scooters)<br>☐ Twirling items (e.g. ribbons, scarves, batons, hula hoops)<br>☐ Throwing & catching items (e.g. balls, beanbags)<br>☐ Balancing items (e.g. balance beams, wood logs or stumps)<br>☐ Crawling or tumbling items (e.g. mats, portable tunnels)<br>☐ Sensory items (e.g. water or sand table)<br>☐ Tables (e.g. for art, resting or eating)<br>☐ Natural play elements (e.g. rain catchers, water troughs)<br>☐ Other (please specify)
Other (please specify)	__________________________
Which of the following types of fixed equipment is available for infant and toddler-aged children to regularly use outdoors?<br>[select all that apply]	☐ Slide<br>☐ Climbing wall or ladder<br>☐ Swings<br>☐ Logs or stumps<br>☐ Sandbox<br>☐ Treehouse<br>☐ Water trough, spout or hose<br>☐ Other (please specify)
Other (please specify)	__________________________
Which of the following types of loose parts are available for infant and toddler-aged children to regularly use outdoors?<br>[select all that apply]	☐ Natural parts (e.g. sticks, branches, leaves, rocks)<br>☐ Construction items (e.g. wood planks, tires, stumps, nails, screws, blocks)<br>☐ Art items (e.g. beads, feathers, shells)<br>☐ Imaginative items (e.g. scarves, dress-up clothing, kites)<br>☐ Sand play items (e.g. shovels, buckets)<br>☐ Gardening tools (e.g. water pots, rakes, spades)<br>☐ Cooking items (pots, pans, spatulas, spoons)<br>☐ Other (please specify)
Other (please specify)	__________________________
What types of gardening areas are accessible to infant and toddler-aged children in your outdoor play area?<br>[select all that apply]	☐ Raised planter beds<br>☐ Garden areas in ground soil<br>☐ Hanging gardening baskets or pots<br>☐ Vines that grow across trellises<br>☐ No gardening areas<br>☐ Other (please specify)
Other (please specify)	__________________________

What types of natural elements are present in your infant/toddler outdoor play area?
[select all that apply]

- ☐ Large trees
- ☐ Small trees
- ☐ Shrubs, bushes, or hedges
- ☐ Flower plants
- ☐ Vegetable plants
- ☐ Wood logs or stumps
- ☐ Other (please specify)

Other (please specify) ________________________________

How often do infant and toddler-aged children at your program have opportunities to do the following activities outdoors?

	Not Allowed	Never	Sometimes	Often	Always
Play at heights or elevation (e.g. climb trees)	○	○	○	○	○
Play with tools (e.g. use power tools, hammer or saws)	○	○	○	○	○
Use fire (e.g. build a campfire under supervision)	○	○	○	○	○
Play with natural elements (e.g. water or leaves)	○	○	○	○	○
Rough and tumble play (e.g. play wrestling, pretend sword fighting)	○	○	○	○	○
Vigorous gross motor play (e.g. walking or running)	○	○	○	○	○
Dramatic, musical, artistic or creative play (e.g. drawing, singing, performing)	○	○	○	○	○
Digital technology play (e.g. iPad, cameras, video)	○	○	○	○	○
Games with rules (e.g. tag, soccer, hide and seek)	○	○	○	○	○
Quiet or restorative play (e.g. reading, storytelling, observing)	○	○	○	○	○
Language, literacy or arithmetic play (e.g. learning numbers or phonetics)	○	○	○	○	○
Water play (e.g. water table or trough)	○	○	○	○	○
Gardening and planting (e.g. seed planting, caring for plants)	○	○	○	○	○
Sand play (e.g. sandbox or sand table)	○	○	○	○	○

Eating (e.g. eating lunch or snack outside)	◯	◯	◯	◯	◯

Indicate how often you take your infant and toddler-aged program to places that are not part of your licensed outdoor play area:

	Daily	2-4 times per week	About 1 time per week	About monthly	Rarely or never
Public playground or park	◯	◯	◯	◯	◯
Private playground or park	◯	◯	◯	◯	◯
Forest	◯	◯	◯	◯	◯
Beach	◯	◯	◯	◯	◯
Wildlife or heritage area	◯	◯	◯	◯	◯
Farm	◯	◯	◯	◯	◯
Other	◯	◯	◯	◯	◯

Other (please specify) ________________________________

In describing your infant/toddler program's curriculum or guiding philosophy, please choose the most accurate descriptor(s):
[select all that apply]

- ☐ Montessori
- ☐ Reggio Emilia
- ☐ Waldorf
- ☐ Emergent
- ☐ Forest School
- ☐ Nature-based education
- ☐ High Scope
- ☐ Froebel
- ☐ Self-developed
- ☐ Other

Other (please specify) ________________________________

Does this infant and toddler-aged program provide all-weather gear or attire for children and/or staff who may not have any at home (e.g. Muddy Buddies, snow suits, boots or ponchos)?
[select one]

- ◯ Yes, for children and staff
- ◯ Yes, for children
- ◯ Yes, for staff
- ◯ No

Please complete the survey below.

Thank you!

Preschool-Aged Children Responses:

Please respond to the following questions regarding the licensed, full-day child care program that serves preschool-aged children.

How many preschool-aged spaces is this licensed, full-day program licensed for (ie. licensed capacity)? ________________________________

[number entry]

How many preschool-aged children are enrolled full-time, full-day in this program? ________________________________
[number entry]

How many preschool-aged children are enrolled part-time or part-day in this program? ________________________________
[number entry]

If this program has a licensed capacity that requires further description, please let us know here.
[text entry] ____

How many preschool-aged children in this program have a diagnosed disability? ________________________________
[number entry]

How many preschool-aged children in this program have a primary language that is not English or French? ________________________________
[number entry]

How many preschool-aged children and families in this program identify as Indigenous (First Nations, Métis, or Inuit)? ________________________________
[number entry]

In a typical week in each season, how many days are preschool-aged children taken outdoors?

	0 days	1 day	2 days	3 days	4 days	5 or more days
Fall	○	○	○	○	○	○
Winter	○	○	○	○	○	○
Spring	○	○	○	○	○	○

Summer	○	○	○	○	○	○

On a typical day in each season, approximately how many hours does each preschool-aged child spend outdoors?

	None	1 hour	1-2 hours	2-3 hours	3-4 hours	5 or more hours
Fall	○	○	○	○	○	○
Winter	○	○	○	○	○	○
Spring	○	○	○	○	○	○
Summer	○	○	○	○	○	○

On a typical day, how is time for outdoor play usually allocated within this preschool-aged program?
[select one]

- ○ According to a fixed schedule
- ○ Spontaneously by the educators
- ○ Mix of scheduled and spontaneous

On a typical day, what is the usual group composition for outdoor play in this preschool-aged program?
[select one]

- ○ Children go outside in one small group
- ○ Children go outside in two small groups
- ○ Children go outside in one large group
- ○ Children go outside in both small groups and as a large group
- ○ Other (please indicate)

Other (please indicate)

____

How often are children able to decide when they want to go outside in this preschool-aged program?
[select one]

- ○ Often/always
- ○ Sometimes/occasionally
- ○ Rarely/never

(Examples - Often/ always: If a child does not want to go outside, they do not have to; or if a child wishes to stay outside longer, they are allowed to. Rarely/ never: Child does not determine when they go outside - this may be set by staff ratios/availability or the schedule for the day.)

How often are preschool-aged children taken outdoors at the following times of the day in the summer months (June - September)?

	Never	Rarely	Occasionally	Often	Always
Early morning (before 10:00am)	○	○	○	○	○
Late morning (10:00am - 12:00pm)	○	○	○	○	○
Afternoon (12:00pm - 2:00pm)	○	○	○	○	○
Late afternoon (2:00pm - 4:00pm)	○	○	○	○	○
Evening (after 4:00pm)	○	○	○	○	○

How often are preschool-aged children taken outdoors at the following times of the day in the winter months (December - March)?

	Never	Rarely	Occasionally	Often	Always
Early morning (before 10:00am)	◯	◯	◯	◯	◯
Late morning (10:00am - 12:00pm)	◯	◯	◯	◯	◯
Afternoon (12:00pm - 2:00pm)	◯	◯	◯	◯	◯
Late afternoon (2:00pm - 4:00pm)	◯	◯	◯	◯	◯
Evening (after 4:00pm)	◯	◯	◯	◯	◯

In your opinion, how important are the following factors in determining how much time preschool-aged children spend outdoors in this program?

	Not Important	Neutral	Important	Very Important	Not Applicable
Educator professional development and training to support outdoor play	◯	◯	◯	◯	◯
Educators are interested in being outdoors and supporting outdoor play	◯	◯	◯	◯	◯
Staffing resources and availability of staff to spend time outdoors	◯	◯	◯	◯	◯
Families support their children playing outside	◯	◯	◯	◯	◯
Size and quality of outdoor play area	◯	◯	◯	◯	◯
Availability of the outdoor play area (e.g. must share space with other programs)	◯	◯	◯	◯	◯
Weather patterns and/or availability of weather-appropriate attire for children and staff	◯	◯	◯	◯	◯
Licensing regulations and requirements	◯	◯	◯	◯	◯
Support for children who require extra support or may exhibit challenging behaviours	◯	◯	◯	◯	◯

Are there other factors that influence how much time children spend outdoors in this program (please indicate)? ________________________________

Do children in this preschool-aged program access the licensed outdoor play area directly from the licensed indoor area?
[select one]

◯ Yes
◯ No

Does this preschool-aged program currently receive any amendments, variances or exemptions to licensing requirements to meet licensing regulations for outdoor play?<br>[select one]	◯ Yes<br>◯ No
Yes (please describe)	__________________________________
Is there an outdoor space that is off-site that is used to meet licensing requirements for this preschool-aged program?<br>[select one]	◯ Yes<br>◯ No
What type of off-site outdoor space is used to meet licensing requirements?	__________________________________
On average, how many minutes does it take preschool-aged children in this program to walk from the indoor space to the off-site outdoor space that is used to meet licensing requirements?<br>[number entry]	__________________________________
Approximately how big is this preschool-aged program's outdoor play area?<br>[select one]	◯ Less than required by licensing regulations (sq. m/child)<br>◯ Exactly what is required by licensing regulations (sq. m/child)<br>◯ Slightly larger than what is required by licensing regulations (sq. m/child)<br>◯ Much larger than what is required by licensing regulations (sq. m/child)
If you are able, please provide the approximate size (square metres) of the outdoor play area that is used by this preschool-aged program.<br>[number entry]	__________________________________<br>(Square metres (approximate))
On the sunniest days of the year, what is the proportion of your outdoor play area for preschool-aged children that receives shade coverage by structures or trees?<br>[select one]	◯ No shade<br>◯ 25% to 50% is shaded<br>◯ 50% to 75% is shaded<br>◯ More than 75% is shaded
Does the preschool outdoor play area have a surrounding fence?<br>[select one]	◯ Yes<br>◯ No

What types of surfacing does the preschool outdoor play area have?<br>[select all that apply]	☐ Artificial grass<br>☐ Bark mulch or engineered wood fibre<br>☐ Grass<br>☐ Concrete<br>☐ Paving stones or tiles<br>☐ Rubber<br>☐ Sand<br>☐ Pea gravel<br>☐ Decking<br>☐ Pebbles<br>☐ Asphalt<br>☐ Dirt<br>☐ Mud<br>☐ Other (please specify)
Other (please specify)	__________________________
Which of following types of portable equipment and materials are available for preschool-aged children to regularly use outdoors?<br>[select all that apply]	☐ Jumping items (e.g. jump ropes, jumping balls)<br>☐ Push-pull items (e.g. wagons, wheelbarrows, big dump trucks)<br>☐ Ride on items (e.g. tricycles, scooters)<br>☐ Twirling items (e.g. ribbons, scarves, batons, hula hoops)<br>☐ Throwing & catching items (e.g. balls, beanbags)<br>☐ Balancing items (e.g. balance beams, wood logs or stumps)<br>☐ Crawling or tumbling items (e.g. mats, portable tunnels)<br>☐ Sensory items (e.g. water or sand table)<br>☐ Tables (e.g. for art, resting or eating)<br>☐ Natural play elements (e.g. rain catchers, water troughs)<br>☐ Other (please specify)
Other (please specify)	__________________________
Which of the following types of fixed equipment is available for preschool-aged children to regularly use outdoors?<br>[select all that apply]	☐ Slide<br>☐ Climbing wall or ladder<br>☐ Swings<br>☐ Logs or stumps<br>☐ Sandbox<br>☐ Treehouse<br>☐ Water trough, spout or hose<br>☐ Other (please specify)
Other (please specify)	__________________________

Which of the following types of loose parts are available for preschool-aged children to regularly use outdoors? [select all that apply]	☐ Natural parts (e.g. sticks, branches, leaves, rocks)<br>☐ Construction items (e.g. wood planks, tires, stumps, nails, screws, blocks)<br>☐ Art items (e.g. beads, feathers, shells)<br>☐ Imaginative items (e.g. scarves, dress-up clothing, kites)<br>☐ Sand play items (e.g. shovels, buckets)<br>☐ Gardening tools (e.g. water pots, rakes, spades)<br>☐ Cooking items (pots, pans, spatulas, spoons)<br>☐ Other (please specify)
Other (please specify)	__________________________
What types of gardening areas are accessible to preschool-aged children in your outdoor play area? [select all that apply]	☐ Raised planter beds<br>☐ Garden areas in ground soil<br>☐ Hanging gardening baskets or pots<br>☐ Vines that grow across trellises<br>☐ No gardening areas<br>☐ Other
Other (please specify)	__________________________
What types of natural elements are present in your preschool outdoor play area? [select all that apply]	☐ Large trees<br>☐ Small trees<br>☐ Shrubs, bushes, or hedges<br>☐ Flower plants<br>☐ Vegetable plants<br>☐ Wood logs or stumps<br>☐ Other (please specify)
Other (please specify)	__________________________

How often do preschool-aged children at your program have opportunities to do the following activities outdoors?

	Not Allowed	Never	Sometimes	Often	Always
Play at heights or elevation (e.g. climb trees)	○	○	○	○	○
Play with tools (e.g. use power tools, hammer or saws)	○	○	○	○	○
Use fire (e.g. build a campfire under supervision)	○	○	○	○	○
Play with natural elements (e.g. water or leaves)	○	○	○	○	○
Rough and tumble play (e.g. play wrestling, pretend sword fighting)	○	○	○	○	○
Vigorous gross motor play (e.g. walking or running)	○	○	○	○	○

Dramatic, musical, artistic or creative play (e.g. drawing, singing, performing)	◯	◯	◯	◯	◯
Digital technology play (e.g. iPad, cameras, video)	◯	◯	◯	◯	◯
Games with rules (e.g. tag, soccer, hide and seek)	◯	◯	◯	◯	◯
Quiet or restorative play (e.g. reading, storytelling, observing)	◯	◯	◯	◯	◯
Language, literacy or arithmetic play (e.g. learning numbers or phonetics)	◯	◯	◯	◯	◯
Water play (e.g. water table or trough)	◯	◯	◯	◯	◯
Gardening and planting (e.g. seed planting, caring for plants)	◯	◯	◯	◯	◯
Sand play (e.g. sandbox or sand table)	◯	◯	◯	◯	◯
Eating (e.g. eating lunch or snack outside)	◯	◯	◯	◯	◯

Indicate how often you take your preschool-aged program to places that are not part of your licensed outdoor play area:

	Daily	2-4 times per week	About 1 time per week	About monthly	Rarely or never
Public playground or park	◯	◯	◯	◯	◯
Private playground or park	◯	◯	◯	◯	◯
Forest	◯	◯	◯	◯	◯
Beach	◯	◯	◯	◯	◯
Wildlife or heritage area	◯	◯	◯	◯	◯
Farm	◯	◯	◯	◯	◯
Other	◯	◯	◯	◯	◯

Other (please specify) ______________________________

In describing your preschool-aged program's curriculum or guiding philosophy, please choose the most accurate descriptor(s):
[select all that apply]

- ☐ Montessori
- ☐ Reggio Emilia
- ☐ Waldorf
- ☐ Emergent
- ☐ Forest School
- ☐ Nature-based education
- ☐ High Scope
- ☐ Froebel
- ☐ Self-developed
- ☐ Other

Other (please specify) ______________________________

Does this preschool-aged program provide all-weather gear or attire for children and/or staff who may not have any at home (e.g. Muddy Buddies, snow suits, boots or ponchos)?
[select one]

- ◯ Yes, for children and staff
- ◯ Yes, for children
- ◯ Yes, for staff
- ◯ No

Infant and Toddler-Aged AND Preschool-Aged Children Responses:

Please respond to the following questions regarding the licensed, full-day child care program that serves preschool-aged children, and the licensed, full-day child care program that serves infant and toddler-aged children.

The following questions refer to your licensed, full-day child care program that serves infant and toddler-aged children.

How many infant and toddler-aged spaces is this licensed, full-day program licensed for (ie. licensed capacity)?
[number entry] ________________________________

How many infant and toddler-aged children are enrolled full-time, full-day in this program?
[number entry] ________________________________

How many infant and toddler-aged children are enrolled part-time or part-day in this program?
[number entry] ________________________________

If this infant and toddler-aged program has a licensed capacity that requires further description, please let us know here.
[text entry] ____

How many infant and toddler-aged children in this program have a diagnosed disability?
[number entry] ________________________________

How many infant and toddler-aged children in this program have a primary language that is not English or French?
[number entry] ________________________________

How many infant and toddler-aged children and families in this program identify as Indigenous (First Nations, Métis, or Inuit)?
[number entry] ________________________________

The following questions refer to your licensed, full-day child care program that serves preschool-aged children.

How many preschool-aged spaces is this licensed, full-day program licensed for (ie. licensed capacity)?

[number entry] ________________________________

How many preschool-aged children are enrolled full-time, full-day in this program?
[number entry] __________________________________

How many preschool-aged children are enrolled part-time or part-day in this program?
[number entry] __________________________________

If this preschool-aged program has a licensed capacity that requires further description, please let us know here.
[text entry] ____

How many preschool-aged children in this program have a diagnosed disability?
[number entry] __________________________________

How many preschool-aged children in this program have a primary language that is not English or French?
[number entry] __________________________________

How many preschool-aged children and families in this program identify as Indigenous (First Nations, Métis, or Inuit)?
[number entry] __________________________________

The following questions refer to your licensed, full-day child care program that serves infant and toddler-aged children.

In a typical week in each season, how many days are infant and toddler-aged children taken outdoors?

	0 days	1 day	2 days	3 days	4 days	5 or more days
Fall	◯	◯	◯	◯	◯	◯
Winter	◯	◯	◯	◯	◯	◯
Spring	◯	◯	◯	◯	◯	◯
Summer	◯	◯	◯	◯	◯	◯

On a typical day in each season, approximately how many hours does each infant and toddler-aged child spend outdoors?

	None	Under 1 hour	1-2 hours	2-3 hours	3-4 hours	5 or more hours
Fall	◯	◯	◯	◯	◯	◯
Winter	◯	◯	◯	◯	◯	◯
Spring	◯	◯	◯	◯	◯	◯
Summer	◯	◯	◯	◯	◯	◯

On a typical day, how is time for outdoor play usually allocated within this infant and toddler-aged program?
[select one]

◯ According to a fixed schedule
◯ Spontaneously by the educators
◯ Mix of scheduled and spontaneous

On a typical day, what is the usual group composition for outdoor play in this infant and toddler-aged program? [select one]	◯ Children go outside in one small group<br>◯ Children go outside in two small groups<br>◯ Children go outside in one large group<br>◯ Children go outside in both small groups and as a large group<br>◯ Other (please indicate)
Other (please indicate)	__________________________________
How often are children able to decide when they want to go outside in this infant and toddler-aged program? [select one]	◯ Often/always<br>◯ Sometimes/occasionally<br>◯ Rarely/never<br>(Examples - Often/ always: If a child does not want to go outside, they do not have to; or if a child wishes to stay outside longer, they are allowed to. Rarely/ never: Child does not determine when they go outside - this may be set by staff ratios/availability or the schedule for the day.)

How often are infant and toddler-aged children taken outdoors at the following times of the day in the summer months (June - September)?

	Never	Rarely	Occasionally	Often	Always
Early morning (before 10:00am)	◯	◯	◯	◯	◯
Late morning (10:00am - 12:00pm)	◯	◯	◯	◯	◯
Afternoon (12:00pm - 2:00pm)	◯	◯	◯	◯	◯
Late afternoon (2:00pm - 4:00pm)	◯	◯	◯	◯	◯
Evening (after 4:00pm)	◯	◯	◯	◯	◯

How often are infant and toddler-aged children taken outdoors at the following times of the day in the winter months (December - March)?

	Never	Rarely	Occasionally	Often	Always
Early morning (before 10:00am)	◯	◯	◯	◯	◯
Late morning (10:00am - 12:00pm)	◯	◯	◯	◯	◯
Afternoon (12:00pm - 2:00pm)	◯	◯	◯	◯	◯
Late afternoon (2:00pm - 4:00pm)	◯	◯	◯	◯	◯
Evening (after 4:00pm)	◯	◯	◯	◯	◯

The following questions refer to your licensed, full-day child care program that serves preschool-aged children.

In a typical week in each season, how many days are preschool-aged children taken outdoors?

	0 days	1 day	2 days	3 days	4 days	5 or more days
Fall	◯	◯	◯	◯	◯	◯

Winter	○	○	○	○	○	○
Spring	○	○	○	○	○	○
Summer	○	○	○	○	○	○

On a typical day in each season, approximately how many hours does each preschool-aged child spend outdoors?

	None	Under 1 hour	1-2 hours	2-3 hours	3-4 hours	5 or more hours
Fall	○	○	○	○	○	○
Winter	○	○	○	○	○	○
Spring	○	○	○	○	○	○
Summer	○	○	○	○	○	○

On a typical day, how is time for outdoor play usually allocated within this preschool-aged program? [select one]

- ○ According to a fixed schedule
- ○ Spontaneously by the educators
- ○ Mix of scheduled and spontaneous

On a typical day, what is the usual group composition for outdoor play in this preschool-aged program? [select one]

- ○ Children go outside in one small group
- ○ Children go outside in two small groups
- ○ Children go outside in one large group
- ○ Children go outside in both small groups and as a large group
- ○ Other (please indicate)

Other (please indicate) ______________________

How often are children able to decide when they want to go outside in this preschool-aged program? [select one]

- ○ Often/always
- ○ Sometimes/occasionally
- ○ Rarely/never

How often are preschool-aged children taken outdoors at the following times of the day in the summer months (June - September)?

	Never	Rarely	Occasionally	Often	Always
Early morning (before 10:00am)	○	○	○	○	○
Late morning (10:00am - 12:00pm)	○	○	○	○	○
Afternoon (12:00pm - 2:00pm)	○	○	○	○	○
Late afternoon (2:00pm - 4:00pm)	○	○	○	○	○
Evening (after 4:00pm)	○	○	○	○	○

How often are preschool-aged children taken outdoors at the following times of the day in the winter months (December - March)?

	Never	Rarely	Occasionally	Often	Always

Early morning (before 10:00am)	○	○	○	○	○
Late morning (10:00am - 12:00pm)	○	○	○	○	○
Afternoon (12:00pm - 2:00pm)	○	○	○	○	○
Late afternoon (2:00pm - 4:00pm)	○	○	○	○	○
Evening (after 4:00pm)	○	○	○	○	○

In your opinion, how important are the following factors in determining how much time infant and toddler-aged children and preschool-aged children spend outdoors in these programs?

	Not Important	Neutral	Important	Very Important	Not Applicable
Educator professional development and training to support outdoor play	○	○	○	○	○
Educators are interested in being outdoors and supporting outdoor play	○	○	○	○	○
Staffing resources and availability of staff to spend time outdoors	○	○	○	○	○
Families support their children playing outside	○	○	○	○	○
Size and quality of outdoor play area	○	○	○	○	○
Availability of the outdoor play area (e.g. must share space with other programs)	○	○	○	○	○
Weather patterns and/or availability of weather-appropriate attire for children and staff	○	○	○	○	○
Licensing regulations and requirements	○	○	○	○	○
Support for children who require extra support or may exhibit challenging behaviours	○	○	○	○	○

Are there other factors that influence how much time children spend outdoors in this program (please indicate)? ______________________________

The following questions refer to your licensed, full-day child care program that serves infant and toddler-aged children.

Do children in the infant and toddler-aged program access the licensed outdoor play area directly from the licensed indoor area?
[select one]

○ Yes
○ No

Does the infant and toddler-aged program currently receive any amendments, variances or exemptions to licensing requirements to meet licensing regulations for outdoor play?<br>[select one]	◯ Yes<br>◯ No
Yes (please describe)	____________________________________
Is there an outdoor space that is off-site that is used to meet licensing requirements for the infant and toddler-aged program?<br>[select one]	◯ Yes<br>◯ No
What type of off-site outdoor space is used to meet licensing requirements?	____________________________________
On average, how many minutes does it take children in the infant and toddler-aged program to walk from the indoor space to the off-site outdoor space that is used to meet licensing requirements?<br>[number entry]	____________________________________
Approximately how big is the infant and toddler-aged program's outdoor play area?<br>[select one]	◯ Less than required by licensing regulations (sq. m/child)<br>◯ Exactly what is required by licensing regulations (sq. m/child)<br>◯ Slightly larger than what is required by licensing regulations (sq. m/child)<br>◯ Much larger than what is required by licensing regulations (sq. m/child)
If you are able, please provide the approximate size (square metres) of the outdoor play area that is used by the infant and toddler-aged program.<br>[number entry]	____________________________________<br>(Square metres (approximate))
On the sunniest days of the year, what is the proportion of your outdoor play area for infant and toddler-aged children that receives shade coverage by structures or trees?<br>[select one]	◯ No shade<br>◯ 25% to 50% is shaded<br>◯ 50% to 75% is shaded<br>◯ More than 75% is shaded
Does the infant and toddler-aged outdoor play area have a surrounding fence?<br>[select one]	◯ Yes<br>◯ No

The following questions refer to your licensed, full-day child care program that serves preschool-aged children.

Do children in the preschool-aged program access the licensed outdoor play area directly from the licensed indoor area?<br>[select one]	◯ Yes<br>◯ No

Does the preschool-aged program currently receive any amendments, variances or exemptions to licensing requirements to meet licensing regulations for outdoor play?<br>[select one]	◯ Yes<br>◯ No
Yes (please describe)	__________________________________
Is there an outdoor space that is off-site that is used to meet licensing requirements for the preschool-aged program?<br>[select one]	◯ Yes<br>◯ No
What type of off-site outdoor space is used to meet licensing requirements?	__________________________________
On average, how many minutes does it take children in the preschool-aged program to walk from the indoor space to the off-site outdoor space that is used to meet licensing requirements?<br>[number entry]	__________________________________
Approximately how big is the preschool-aged program's outdoor play area?<br>[select one]	◯ Less than required by licensing regulations (sq. m/child)<br>◯ Exactly what is required by licensing regulations (sq. m/child)<br>◯ Slightly larger than what is required by licensing regulations (sq. m/child)<br>◯ Much larger than what is required by licensing regulations (sq. m/child)
If you are able, please provide the approximate size (square metres) of the outdoor play area that is used by the preschool-aged program.<br>[number entry]	__________________________________<br>(Square metres (approximate))
On the sunniest days of the year, what is the proportion of your outdoor play area for preschool-aged children that receives shade coverage by structures or trees?<br>[select one]	◯ No shade<br>◯ 25% to 50% is shaded<br>◯ 50% to 75% is shaded<br>◯ More than 75% is shaded
Does the preschool-aged outdoor play area have a surrounding fence?<br>[select one]	◯ Yes<br>◯ No

The following questions refer to your licensed, full-day child care program that serves infant and toddler-aged children.

What types of surfacing does the infant and toddler-aged outdoor play area have?<br>[select all that apply]	☐ Artificial grass<br>☐ Bark mulch or engineered wood fibre<br>☐ Grass<br>☐ Concrete<br>☐ Paving stones or tiles<br>☐ Rubber<br>☐ Sand<br>☐ Pea gravel<br>☐ Decking<br>☐ Pebbles<br>☐ Asphalt<br>☐ Dirt<br>☐ Mud<br>☐ Other (please specify)
Other (please specify)	__________________________________
Which of following types of portable equipment and materials are available for infant and toddler-aged children to regularly use outdoors?<br>[select all that apply]	☐ Jumping items (e.g. jump ropes, jumping balls)<br>☐ Push-pull items (e.g. wagons, wheelbarrows, big dump trucks)<br>☐ Ride on items (e.g. tricycles, scooters)<br>☐ Twirling items (e.g. ribbons, scarves, batons, hula hoops)<br>☐ Throwing & catching items (e.g. balls, beanbags)<br>☐ Balancing items (e.g. balance beams, wood logs or stumps)<br>☐ Crawling or tumbling items (e.g. mats, portable tunnels)<br>☐ Sensory items (e.g. water or sand table)<br>☐ Tables (e.g. for art, resting or eating)<br>☐ Natural play elements (e.g. rain catchers, water troughs)<br>☐ Other (please specify)
Other (please specify)	__________________________________
Which of the following types of fixed equipment is available for infant and toddler-aged children to regularly use outdoors?<br>[select all that apply]	☐ Slide<br>☐ Climbing wall or ladder<br>☐ Swings<br>☐ Logs or stumps<br>☐ Sandbox<br>☐ Treehouse<br>☐ Water trough, spout or hose<br>☐ Other (please specify)
Other (please specify)	__________________________________

Which of the following types of loose parts are available for infant and toddler-aged children to regularly use outdoors?<br>[select all that apply]	☐ Natural parts (e.g. sticks, branches, leaves, rocks)<br>☐ Construction items (e.g. wood planks, tires, stumps, nails, screws, blocks)<br>☐ Art items (e.g. beads, feathers, shells)<br>☐ Imaginative items (e.g. scarves, dress-up clothing, kites)<br>☐ Sand play items (e.g. shovels, buckets)<br>☐ Gardening tools (e.g. water pots, rakes, spades)<br>☐ Cooking items (pots, pans, spatulas, spoons)<br>☐ Other (please specify)
Other (please specify)	______________________________
What types of gardening areas are accessible to infant and toddler-aged children in your outdoor play area?<br>[select all that apply]	☐ Raised planter beds<br>☐ Garden areas in ground soil<br>☐ Hanging gardening baskets or pots<br>☐ Vines that grow across trellises<br>☐ No gardening areas<br>☐ Other (please specify)
Other (please specify)	______________________________
What types of natural elements are present in your infant and toddler-aged outdoor play area?<br>[select all that apply]	☐ Large trees<br>☐ Small trees<br>☐ Shrubs, bushes, or hedges<br>☐ Flower plants<br>☐ Vegetable plants<br>☐ Wood logs or stumps<br>☐ Other (please specify)
Other (please specify)	______________________________

The following questions refer to your licensed, full-day child care program that serves preschool-aged children.

What types of surfacing does the preschool-aged outdoor play area have?<br>[select all that apply]	☐ Artificial grass<br>☐ Bark mulch or engineered wood fibre<br>☐ Grass<br>☐ Concrete<br>☐ Paving stones or tiles<br>☐ Rubber<br>☐ Sand<br>☐ Pea gravel<br>☐ Decking<br>☐ Pebbles<br>☐ Asphalt<br>☐ Dirt<br>☐ Mud<br>☐ Other (please specify)
Other (please specify)	______________________________

Which of following types of portable equipment and materials are available for preschool-aged children to regularly use outdoors?<br>[select all that apply]	☐ Jumping items (e.g. jump ropes, jumping balls)<br>☐ Push-pull items (e.g. wagons, wheelbarrows, big dump trucks)<br>☐ Ride on items (e.g. tricycles, scooters)<br>☐ Twirling items (e.g. ribbons, scarves, batons, hula hoops)<br>☐ Throwing & catching items (e.g. balls, beanbags)<br>☐ Balancing items (e.g. balance beams, wood logs or stumps)<br>☐ Crawling or tumbling items (e.g. mats, portable tunnels)<br>☐ Sensory items (e.g. water or sand table)<br>☐ Tables (e.g. for art, resting or eating)<br>☐ Natural play elements (e.g. rain catchers, water troughs)<br>☐ Other (please specify)
Other (please specify)	__________________________________
Which of the following types of fixed equipment is available for preschool-aged children to regularly use outdoors?<br>[select all that apply]	☐ Slide<br>☐ Climbing wall or ladder<br>☐ Swings<br>☐ Logs or stumps<br>☐ Sandbox<br>☐ Treehouse<br>☐ Water trough, spout or hose<br>☐ Other (please specify)
Other (please specify)	__________________________________
Which of the following types of loose parts are available for preschool-aged children to regularly use outdoors?<br>[select all that apply]	☐ Natural parts (e.g. sticks, branches, leaves, rocks)<br>☐ Construction items (e.g. wood planks, tires, stumps, nails, screws, blocks)<br>☐ Art items (e.g. beads, feathers, shells)<br>☐ Imaginative items (e.g. scarves, dress-up clothing, kites)<br>☐ Sand play items (e.g. shovels, buckets)<br>☐ Gardening tools (e.g. water pots, rakes, spades)<br>☐ Cooking items (pots, pans, spatulas, spoons)<br>☐ Other (please specify)
Other (please specify)	__________________________________
What types of gardening areas are accessible to preschool-aged children in your outdoor play area?<br>[select all that apply]	☐ Raised planter beds<br>☐ Garden areas in ground soil<br>☐ Hanging gardening baskets or pots<br>☐ Vines that grow across trellises<br>☐ No gardening areas<br>☐ Other (please specify)
Other (please specify)	__________________________________

What types of natural elements are present in your preschool-aged outdoor play area?
[select all that apply]

- ☐ Large trees
- ☐ Small trees
- ☐ Shrubs, bushes, or hedges
- ☐ Flower plants
- ☐ Vegetable plants
- ☐ Wood logs or stumps
- ☐ Other (please specify)

Other (please specify) ______________________________

The following questions refer to your licensed, full-day child care program that serves infant and toddler-aged children.

How often do infant and toddler-aged children at your program have opportunities to do the following activities outdoors?

	Not Allowed	Never	Sometimes	Often	Always
Play at heights or elevation (e.g. climb trees)	○	○	○	○	○
Play with tools (e.g. use power tools, hammer or saws)	○	○	○	○	○
Use fire (e.g. build a campfire under supervision)	○	○	○	○	○
Play with natural elements (e.g. water or leaves)	○	○	○	○	○
Rough and tumble play (e.g. play wrestling, pretend sword fighting)	○	○	○	○	○
Vigorous gross motor play (e.g. walking or running)	○	○	○	○	○
Dramatic, musical, artistic or creative play (e.g. drawing, singing, performing)	○	○	○	○	○
Digital technology play (e.g. iPad, cameras, video)	○	○	○	○	○
Games with rules (e.g. tag, soccer, hide and seek)	○	○	○	○	○
Quiet or restorative play (e.g. reading, storytelling, observing)	○	○	○	○	○
Language, literacy or arithmetic play (e.g. learning numbers or phonetics)	○	○	○	○	○
Water play (e.g. water table or trough)	○	○	○	○	○
Gardening and planting (e.g. seed planting, caring for plants)	○	○	○	○	○

Sand play (e.g. sandbox or sand table)	◯	◯	◯	◯	◯
Eating (e.g. eating lunch or snack outside)	◯	◯	◯	◯	◯

Indicate how often you take your infant and toddler-aged program to places that are not part of your licensed outdoor play area:

	Daily	2-4 times per week	About 1 time per week	About monthly	Rarely or never
Public playground or park	◯	◯	◯	◯	◯
Private playground or park	◯	◯	◯	◯	◯
Forest	◯	◯	◯	◯	◯
Beach	◯	◯	◯	◯	◯
Wildlife or heritage area	◯	◯	◯	◯	◯
Farm	◯	◯	◯	◯	◯
Other	◯	◯	◯	◯	◯

Other (please specify) ______________________________

The following questions refer to your licensed, full-day child care program that serves preschool-aged children.

How often do preschool-aged children at your program have opportunities to do the following activities outdoors?

	Not Allowed	Never	Sometimes	Often	Always
Play at heights or elevation (e.g. climb trees)	◯	◯	◯	◯	◯
Play with tools (e.g. use power tools, hammer or saws)	◯	◯	◯	◯	◯
Use fire (e.g. build a campfire under supervision)	◯	◯	◯	◯	◯
Play with natural elements (e.g. water or leaves)	◯	◯	◯	◯	◯
Rough and tumble play (e.g. play wrestling, pretend sword fighting)	◯	◯	◯	◯	◯
Vigorous gross motor play (e.g. walking or running)	◯	◯	◯	◯	◯
Dramatic, musical, artistic or creative play (e.g. drawing, singing, performing)	◯	◯	◯	◯	◯
Digital technology play (e.g. iPad, cameras, video)	◯	◯	◯	◯	◯
Games with rules (e.g. tag, soccer, hide and seek)	◯	◯	◯	◯	◯

Quiet or restorative play (e.g. reading, storytelling, observing)	○	○	○	○	○
Language, literacy or arithmetic play (e.g. learning numbers or phonetics)	○	○	○	○	○
Water play (e.g. water table or trough)	○	○	○	○	○
Gardening and planting (e.g. seed planting, caring for plants)	○	○	○	○	○
Sand play (e.g. sandbox or sand table)	○	○	○	○	○
Eating (e.g. eating lunch or snack outside)	○	○	○	○	○

Indicate how often you take your preschool-aged program to places that are not part of your licensed outdoor play area:

	Daily	2-4 times per week	About 1 time per week	About monthly	Rarely or never
Public playground or park	○	○	○	○	○
Private playground or park	○	○	○	○	○
Forest	○	○	○	○	○
Beach	○	○	○	○	○
Wildlife or heritage area	○	○	○	○	○
Farm	○	○	○	○	○
Other	○	○	○	○	○

Other (please specify) __________________________________

The following questions refer to your licensed, full-day child care program that serves infant and toddler-aged children.

In describing your infant and toddler-aged program's curriculum or guiding philosophy, please choose the most accurate descriptor(s):
[select all that apply]

- ☐ Montessori
- ☐ Reggio Emilia
- ☐ Waldorf
- ☐ Emergent
- ☐ Forest School
- ☐ Nature-based education
- ☐ High Scope
- ☐ Froebel
- ☐ Self-developed
- ☐ Other

Other (please specify) ______________________________

Does this infant and toddler-aged program provide all-weather gear or attire for children and/or staff who may not have any at home (e.g. Muddy Buddies, snow suits, boots or ponchos)?
[select one]

- ◯ Yes, for children and staff
- ◯ Yes, for children
- ◯ Yes, for staff
- ◯ No

The following questions refer to your licensed, full-day child care program that serves preschool-aged children.

In describing your preschool-aged program's curriculum or guiding philosophy, please choose the most accurate descriptor(s):
[select all that apply]

- ☐ Montessori
- ☐ Reggio Emilia
- ☐ Waldorf
- ☐ Emergent
- ☐ Forest School
- ☐ Nature-based education
- ☐ High Scope
- ☐ Froebel
- ☐ Self-developed
- ☐ Other

Other (please specify) ______________________________

Does this preschool-aged program provide all-weather gear or attire for children and/or staff who may not have any at home (e.g. Muddy Buddies, snow suits, boots or ponchos)?
[select one]

- ◯ Yes, for children and staff
- ◯ Yes, for children
- ◯ Yes, for staff
- ◯ No

Have you taken any specific training related to outdoor play in early childhood education in the past five years? [select one]	◯ Yes<br>◯ No
Yes (please indicate)	__________________________
Have other staff at this centre taken any specific training related to outdoor play in early childhood education in the past five years? [select one]	◯ Yes<br>◯ No<br>◯ Unsure
How many staff have taken outdoor play-related training in the past five years? [number entry]	__________________________
What types of training, courses or workshops have been taken related to outdoor play? [text entry]	__________________________
Which of the following challenges does this centre currently experience in supporting children of all developmental and physical abilities to participate in outdoor play? [select all that apply]	☐ Outdoor space is not accessible<br>☐ Outdoor equipment and materials aren't appropriate<br>☐ Educators are not comfortable fully supporting child(ren)<br>☐ Educators are not trained to support child(ren)<br>☐ No challenges - all children are able to fully participate in outdoor play<br>☐ Other (please specify)
Other (please specify)	__________________________
How many paid child care staff do you have whose day-to-day responsibility includes working directly with children at this centre? [number entry]	__________________________
Of the staff who work with children, how many have worked at this centre less than 1 year? [number entry]	__________________________
Of the staff who work with children, how many have worked at this centre between 1 and 4 years? [number entry]	__________________________
Of the staff who work with children, how many have worked at this centre 5 or longer? [number entry]	__________________________
Of the staff who work with children, how many have no ECE-specific post-secondary education? [number entry]	__________________________

Of the staff who work with children, how many have ECE-specific workshops or courses, but no ECE-specific credential?
[number entry]

____

Of the staff who work with children, how many have a one-year ECE-specific certificate or diploma?
[number entry]

____

Of the staff who work with children, how many have a two- or three-year ECE-specific diploma?
[number entry]

____

Of the staff who work with children, how many have an ECE-specific Bachelor's degree, Master's degree or Ph.D.?
[number entry]

____

Of the staff who work with children, how many are fluent in a language other than English or French?
[number entry]

____

Of the staff who work with children, how many identify as Indigenous (First Nations, Métis, or Inuit)?
[number entry]

____

How many licensed infant/toddler spaces is your centre licensed for?
[number entry]

____

How many licensed preschool-aged spaces is your centre licensed for?
[number entry]

____

To give us more understanding of your outdoor play space, we welcome you to upload a photo(s) of each program's outdoor space here.

Please upload any photo that shows the full extent of your outdoor space rather than individual elements. You can upload up to 2 photos.

Uploaded files have a maximum size limit of 500MB. To reduce the size, we suggest compressing their photo into a ZIP file first or changing the format of the file to reduce the file size. If you are unable to upload the photos here, please feel free to email them to outdoorplay@bcchr.ca. Please use the same email for your gift card entry.

Photo 1

Photo 2

Please provide a description of your photo(s) indicating the program or space. Is there any other information you wish to provide that describes your outdoor space? ____

Is there anything else you would like to tell us about outdoor play at this child care centre? ____

Redirect to Gift Card

Thank you for completing the survey! Click "Submit" to provide your work email address to be eligible for receiving a $25 Amazon gift card as a thank you for completing our survey.

Secondary ID	________________
Please provide your work email address to be eligible for receiving a $25 Amazon gift card as a thank you for completing our survey.<br><br>Please note that your email address will not be linked to any of the data provided in this survey following the completion of the gift card distribution. Gift cards are available for the first 1,000 respondents who fully complete the survey and verification of responses may be requested by the research team.	________________
Would you be interested in participating in further data collection on outdoor play at child care centres?	◯ Yes<br>◯ No
Is [e10_2] your email address you can be contacted for further participation? If not, please enter your email address you wish to be contacted at for further participation.<br><br>Please note that your email address will not be linked to any of the data provided in this survey.	________________
Would you like to be contacted to receive information about the findings of this study?	◯ Yes<br>◯ No
Is [e10_2] your email address you can be contacted when the findings are available? If not, please enter your email address you wish to be contacted at when the findings are available.<br><br>Please note that your email address will not be linked to any of the data provided in this survey.	________________
