# Supplementary material for: Examining outdoor play associations in Canadian early learning and child care centres: Cross-sectional insights from the Measuring Early Childhood Outside survey
Source: PLoS One. 2026 Feb 11;21(2):e0331166. doi: 10.1371/journal.pone.0331166 (PMC12893580; doi:10.1371/journal.pone.0331166)
Supplement: S2 Appendix — (DOCX) [file pone.0331166.s002.docx]

## S2 Appendix: Selection of Explanatory Variables

| **Explanatory Variable** | **Category** | **Variable Levels** |
| --- | --- | --- |
| Auspice | Centre-level | 2 |
| Urban/Rural Classification | Centre-level | 2 |
| Centre Age Groups | Centre-level | 2 |
| OP Training | Staff-related | 4 |
| Educator Tenure at Centre | Staff-related | 3 |
| Size of OP Area | Physical Environment | 4 |
| Access to OP Area | Physical Environment | 2 |
| Count of Loose Parts | Physical Environment | 9 |
| Count of Gardening Areas | Physical Environment | 5 |
| Count of Natural Elements | Physical Environment | 7 |
| Count of Fixed Equipment Types | Physical Environment | 9 |
| Count of Portable Equipment Types | Physical Environment | 11 |
| Provision of All-Weather Gear | OP Provision | 3 |
| Children’s OP Autonomy | OP Provision | 3 |

## *Centre-level Explanatory Variables*

Three explanatory variables related to centre-level characteristics were included: 1) auspice; 2) rural-urban classification; and 3) centre age groups. These variables were assessed at the centre-level and not by program type, therefore descriptive results are not separated by I/T and PS programs. While respondents were able to indicate their specific mode of operation (e.g. government-run, Indigenous-led, sole proprietor, etc.), responses were dichotomized (non-profit/ for-profit) to facilitate analysis. An urban-rural classification variable was derived from respondents’ postal codes, where rural areas contain a “0” in the second character as per Canada Post postal code conventions. The variable ‘centre age groups’ (I/T only; PS only; both I/T and PS) considered if the centre served one age group or multiple age groups, which is related to the delivery of outdoor play and how it is organized within an ELCC centre.

## *Staff-related Explanatory Variables*

Two explanatory variables related to staff-related characteristics were included: 1) OP training; and 2) educator tenure at the centre. These variables were assessed at the centre-level and not by program type, therefore descriptive results are not separated by I/T and PS programs. Staffing characteristics were assessed for centre directors and educators; educators were defined as professionals that work directly with children at the centre and encompassed multiple terms (early childhood educator, child care worker) and certification levels (child care assistant, early childhood educator assistant) indicative of the regional differences in terminology and education requirements. The variable ‘OP training’ sought to understand which staff had previously received OP training at the responding centre (none, director only, educators only, both director and educators). Educator tenure at the centre was assessed by analyzing the proportion of educators who had worked five years or longer at the responding centre, and reflected a measure of staffing stability. Respondents were asked to indicate, considering all educators who work with children, the number of which have various lengths of time working at the centre. These responses were translated into an ordinal variable indicating low tenure (under 25% of educators had 5+ years at the centre), middle tenure (25% to 49.9% of educators had 5+ years at the centre), or high tenure (50% and over of educators had 5+ years at the centre).

## *Physical Environment Explanatory Variables*

Seven explanatory variables related to physical environment characteristics were included: 1) OP area size; 2) access to OP area; 3) count of loose parts; 4) count of gardening areas; 5) count of natural elements; 6) count of fixed equipment types; and 7) count of portable equipment types. These variables were assessed at the program-level, therefore descriptive results are presented separately for I/T and PS programs. The size of the OP area (less than required, exactly as required, slightly larger than required, much larger than required) was assessed in relation to regional licensing requirements. Centres reporting relying on off-site OP areas to meet licensing requirements were combined with centres responding “less than required” for their on-site outdoor space to facilitate a comprehensive OP area size variable that included all respondents. The variable ‘access to OP area’ allowed respondents to identify if their outdoor space had direct access from their indoor space (yes/no). The counts of loose parts (natural materials, cooking items, etc.), gardening areas (planter beds, hanging baskets, etc.), natural elements (trees, shrubs, etc.), and fixed (slide, sandbox, etc.) and portable (ride on items, tables, etc.) equipment were included as indicators of the affordances for play provided in the spaces. For each of these explanatory variables, a count of available items was calculated, with higher numbers being assumed to indicate more play affordances within that variable.

## *Provision-related Explanatory Variables*

Two explanatory variables related to overall OP provision included in the analysis were: 1) provision of all-weather gear; and 2) children’s OP autonomy. These variables were assessed at the program-level, therefore descriptive results are presented separately for I/T and PS programs. Respondents identified if their program(s) provided all-weather gear for children, educators or both. Children’s play autonomy was assessed by asking respondents to identify how often children are able to decide when they want to go outside (often/always, sometimes/occasionally, rarely/never).
