# Supplementary material for: Examining outdoor play associations in Canadian early learning and child care centres: Cross-sectional insights from the Measuring Early Childhood Outside survey
Source: PLoS One. 2026 Feb 11;21(2):e0331166. doi: 10.1371/journal.pone.0331166 (PMC12893580; doi:10.1371/journal.pone.0331166)
Supplement: S3 Appendix — (DOCX) [file pone.0331166.s003.docx]

# S3 Appendix: Hierarchical Multiple Regression Models for All Outcomes

Table A1: Hierarchical Multiple Regression Models for Summer Duration, Infant/Toddler Programs

|  |  | Summer Duration in I/T Centres: Betas & Significance | | | |
| --- | --- | --- | --- | --- | --- |
|  |  | Only Centre Characteristics | Add Staff Characteristics | Add Physical Environment and Additional Characteristics | Add Interactions |
| Auspice | Non-profit vs. For-profit | -.059 | **-.076*** | **-.127***** | **-.126***** |
| Rural/Urban | Rural vs. Urban Postal Code | .037 | .042 | .017 | .017 |
| Centre Ages | Both Ages vs. Just I/T | **-.087**** | **-.082*** | -.062 | -.058 |
| OP Area Size | Smaller/Off-Site vs. As required | .076 | .07 | .013 | .017 |
|  | Larger vs. As required | .022 | .015 | .019 | .024 |
|  | Much larger vs. As required | **.169***** | **.153**** | **.114*** | **.111*** |
| Access to OP Area | Direct vs. Not | **.099**** | **.097**** | **.071*** | **.071*** |
| OP Training | Director Only vs. None |  | .058 | .05 | .046 |
|  | Educator Only vs. None |  | .06 | .05 | .051 |
|  | Director and Educators vs. None |  | .04 | -.008 | -.009 |
| Staff Tenure | Medium vs. Low |  | .036 | .02 | .016 |
|  | High vs. Low |  | .059 | .055 | .055 |
| All-Weather Gear | Children or Educators vs. Neither |  |  | .058 | .061 |
|  | Children and Educators vs. Neither |  |  | **.076*** | **.076*** |
| Child Autonomy | Medium vs. Low |  |  | **.128***** | **.169***** |
|  | High vs. Low |  |  | **.066*** | **.126*** |
| Affordances | Loose Parts |  |  | **.142***** | **.155**** |
|  | Gardening Elements |  |  | **.097*** | **.096*** |
|  | Natural Elements |  |  | -.041 | -.033 |
|  | Fixed Equipment |  |  | **.153***** | **.119*** |
|  | Portable Equipment |  |  | -.039 | -.035 |
| Interactions | Director Only OP Training x Loose Parts |  |  |  | -.041 |
|  | Educator Only OP Training x Loose Parts |  |  |  | -.006 |
|  | Both with OP Training x Loose Parts |  |  |  | .004 |
|  | Director Only OP Training x Fixed Equipment |  |  |  | .054 |
|  | Educator Only OP Training x Fixed Equipment |  |  |  | .011 |
|  | Both with OP Training x Fixed Equipment |  |  |  | .011 |
|  | Director Only OP Training x Autonomy |  |  |  | .063 |
|  | Educator Only OP Training x Autonomy |  |  |  | -.026 |
|  | Both with OP Training x Autonomy |  |  |  | .079 |
|  |  |  |  |  |  |
|  | Adjusted R^2^ and Significance of R^2^ Change | .040*** | .042 | .135*** | .134 |
|  | N (weighted) | 920 | 920 | 920 | 920 |

* *p*<.05, ** *p*<.01, *** *p*<.001

Table A2: Hierarchical Multiple Regression Models for Summer Duration, Preschool-Age Programs

|  |  | Summer Duration in PS Centres: Betas & Significance | | | |
| --- | --- | --- | --- | --- | --- |
|  |  | Only Centre Characteristics | Add Staff Characteristics | Add Physical Environment and Additional Characteristics | Add Interactions |
| Auspice | Non-profit vs. For-profit | -.037 | **-.067*** | **-.121***** | **-.115***** |
| Rural/Urban | Rural vs. Urban Postal Code | **.082**** | **.090**** | **.063*** | **.060*** |
| Centre Ages | Both Ages vs. Just I/T | .059 | .049 | .029 | .030 |
| OP Area Size | Smaller/Off-Site vs. As required | **.220***** | **.214***** | **.168***** | **.180***** |
|  | Larger vs. As required | **.105*** | **.107*** | .088 | .083 |
|  | Much larger vs. As required | **.286***** | **.280***** | **.218***** | **.216***** |
| Access to OP Area | Direct vs. Not | **.081**** | **.068*** | .014 | .018 |
| OP Training | Director Only vs. None |  | .038 | .004 | -.006 |
|  | Educator Only vs. None |  | **.088**** | **.080*** | **.085**** |
|  | Director and Educators vs. None |  | **.134***** | **.075*** | **.081*** |
| Staff Tenure | Medium vs. Low |  | .025 | .000 | .001 |
|  | High vs. Low |  | .024 | -.002 | -.001 |
| All-Weather Gear | Children or Educators vs. Neither |  |  | .039 | .044 |
|  | Children and Educators vs. Neither |  |  | -.002 | -.006 |
| Child Autonomy | Medium vs. Low |  |  | **.105**** | **.148***** |
|  | High vs. Low |  |  | **.104**** | **.177***** |
| Affordances | Loose Parts |  |  | **.096**** | **.111*** |
|  | Gardening Elements |  |  | .060 | .062 |
|  | Natural Elements |  |  | .043 | .053 |
|  | Fixed Equipment |  |  | **.106**** | .036 |
|  | Portable Equipment |  |  | .053 | .056 |
| Interactions | Director Only OP Training x Loose Parts |  |  |  | -.031 |
|  | Educator Only OP Training x Loose Parts |  |  |  | .028 |
|  | Both with OP Training x Loose Parts |  |  |  | -.011 |
|  | Director Only OP Training x Fixed Equipment |  |  |  | **.118***** |
|  | Educator Only OP Training x Fixed Equipment |  |  |  | -.019 |
|  | Both with OP Training x Fixed Equipment |  |  |  | .029 |
|  | Director Only OP Training x Autonomy |  |  |  | .008 |
|  | Educator Only OP Training x Autonomy |  |  |  | .014 |
|  | Both with OP Training x Autonomy |  |  |  | **.114**** |
|  |  |  |  |  |  |
|  | Adjusted R^2^ and Significance of R^2^ Change | .054*** | .068** | .138*** | .150** |
|  | N (weighted) | 1,028 | 1,028 | 1,028 | 1,028 |

* *p*<.05, ** *p*<.01, *** *p*<.001

Table A3: Hierarchical Multiple Regression Models for Winter Duration, Infant/Toddler Programs

|  |  | Winter Duration in I/T Centres: Betas & Significance | | | |
| --- | --- | --- | --- | --- | --- |
|  |  | Only Centre Characteristics | Add Staff Characteristics | Add Physical Environment and Additional Characteristics | Add Interactions |
| Auspice | Non-profit vs. For-profit | .027 | .019 | -.020 | -.006 |
| Rural/Urban | Rural vs. Urban Postal Code | -.032 | -.029 | -.038 | -.034 |
| Centre Ages | Both Ages vs. Just I/T | -.065 | **-.075*** | **-.075*** | **-.074*** |
| OP Area Size | Smaller/Off-Site vs. As required | .016 | .003 | -.033 | -.030 |
|  | Larger vs. As required | .021 | .009 | .021 | .028 |
|  | Much larger vs. As required | .007 | .000 | -.010 | -.009 |
| Access to OP Area | Direct vs. Not | -.039 | -.038 | -.045 | -.035 |
| OP Training | Director Only vs. None |  | .056 | .044 | .046 |
|  | Educator Only vs. None |  | .039 | .027 | .029 |
|  | Director and Educators vs. None |  | **.094*** | .043 | .048 |
| Staff Tenure | Medium vs. Low |  | -.047 | -.064 | -.067 |
|  | High vs. Low |  | -.049 | -.064 | -.063 |
| All-Weather Gear | Children or Educators vs. Neither |  |  | .036 | .034 |
|  | Children and Educators vs. Neither |  |  | **.107**** | **.097**** |
| Child Autonomy | Medium vs. Low |  |  | .057 | **.102*** |
|  | High vs. Low |  |  | **.090**** | **.161**** |
| Affordances | Loose Parts |  |  | **.153***** | **.127*** |
|  | Gardening Elements |  |  | **.118**** | **.112*** |
|  | Natural Elements |  |  | .046 | .053 |
|  | Fixed Equipment |  |  | -.030 | -.080 |
|  | Portable Equipment |  |  | -.051 | -.047 |
| Interactions | Director Only OP Training x Loose Parts |  |  |  | .030 |
|  | Educator Only OP Training x Loose Parts |  |  |  | 000 |
|  | Both with OP Training x Loose Parts |  |  |  | .025 |
|  | Director Only OP Training x Fixed Equipment |  |  |  | .057 |
|  | Educator Only OP Training x Fixed Equipment |  |  |  | **.106**** |
|  | Both with OP Training x Fixed Equipment |  |  |  | -.015 |
|  | Director Only OP Training x Autonomy |  |  |  | .024 |
|  | Educator Only OP Training x Autonomy |  |  |  | .051 |
|  | Both with OP Training x Autonomy |  |  |  | .083 |
|  |  |  |  |  |  |
|  | Adjusted R^2^ and Significance of R^2^ Change | -.001 | .003 | .076*** | .084* |
|  | N (weighted) | 922 | 922 | 922 | 922 |

* *p*<.05, ** *p*<.01, *** *p*<.001

Table A4: Hierarchical Multiple Regression Models for Winter Duration, Preschool-Age Programs

|  |  | Winter Duration in PS Centres: Betas & Significance | | | |
| --- | --- | --- | --- | --- | --- |
|  |  | Only Centre Characteristics | Add Staff Characteristics | Add Physical Environment and Additional Characteristics | Add Interactions |
| Auspice | Non-profit vs. For-profit | .000 | -.010 | -.058 | -.05 |
| Rural/Urban | Rural vs. Urban Postal Code | -.014 | -.010 | -.026 | -.027 |
| Centre Ages | Both Ages vs. Just I/T | **.091**** | **.088**** | **.099**** | **.098**** |
| OP Area Size | Smaller/Off-Site vs. As required | .023 | .016 | -.027 | -.018 |
|  | Larger vs. As required | .013 | .015 | .008 | .002 |
|  | Much larger vs. As required | .092 | .091 | .075 | .068 |
| Access to OP Area | Direct vs. Not | -.004 | -.012 | -.054 | -.051 |
| OP Training | Director Only vs. None |  | .034 | .015 | .011 |
|  | Educator Only vs. None |  | .001 | -.003 | .006 |
|  | Director and Educators vs. None |  | **.087*** | .023 | .023 |
| Staff Tenure | Medium vs. Low |  | .011 | -.021 | -.017 |
|  | High vs. Low |  | -.015 | -.018 | -.017 |
| All-Weather Gear | Children or Educators vs. Neither |  |  | .056 | **.066*** |
|  | Children and Educators vs. Neither |  |  | -.003 | -.002 |
| Child Autonomy | Medium vs. Low |  |  | **.074*** | **.138***** |
|  | High vs. Low |  |  | **.153***** | **.259***** |
| Affordances | Loose Parts |  |  | **.235***** | **.193***** |
|  | Gardening Elements |  |  | .005 | .014 |
|  | Natural Elements |  |  | **.095*** | **.095*** |
|  | Fixed Equipment |  |  | -.015 | -.069 |
|  | Portable Equipment |  |  | -.051 | -.047 |
| Interactions | Director Only OP Training x Loose Parts |  |  |  | -.013 |
|  | Educator Only OP Training x Loose Parts |  |  |  | .048 |
|  | Both with OP Training x Loose Parts |  |  |  | .056 |
|  | Director Only OP Training x Fixed Equipment |  |  |  | **.082*** |
|  | Educator Only OP Training x Fixed Equipment |  |  |  | .037 |
|  | Both with OP Training x Fixed Equipment |  |  |  | .010 |
|  | Director Only OP Training x Autonomy |  |  |  | .058 |
|  | Educator Only OP Training x Autonomy |  |  |  | .016 |
|  | Both with OP Training x Autonomy |  |  |  | **.139**** |
|  |  |  |  |  |  |
|  | Adjusted R^2^ and Significance of R^2^ Change | .007 | .009 | .101*** | .110* |
|  | N (weighted) | 1,035 | 1,035 | 1,035 | 1,035 |

* *p*<.05, ** *p*<.01, *** *p*<.001

Table A5: Hierarchical Multiple Regression Models for Winter Frequency, Infant/Toddler Programs

|  |  | Winter Frequency in I/T Centres: Betas & Significance | | | |
| --- | --- | --- | --- | --- | --- |
|  |  | Only Centre Characteristics | Add Staff Characteristics | Add Physical Environment and Additional Characteristics | Add Interactions |
| Auspice | Non-profit vs. For-profit | **.080*** | .060 | .027 | .040 |
| Rural/Urban | Rural vs. Urban Postal Code | -.013 | -.004 | -.010 | -.022 |
| Centre Ages | Both Ages vs. Just I/T | -.030 | -.041 | -.042 | -.037 |
| OP Area Size | Smaller/Off-Site vs. As required | .062 | .036 | .028 | .024 |
|  | Larger vs. As required | **.158**** | **.149**** | **.155**** | **.132**** |
|  | Much larger vs. As required | **.120*** | **.108*** | .086 | .068 |
| Access to OP Area | Direct vs. Not | -.019 | -.023 | -.031 | -.034 |
| OP Training | Director Only vs. None |  | .005 | -.003 | -.008 |
|  | Educator Only vs. None |  | -.035 | -.038 | -.039 |
|  | Director and Educators vs. None |  | **.163***** | **.125***** | **.147***** |
| Staff Tenure | Medium vs. Low |  | .004 | -.013 | -.020 |
|  | High vs. Low |  | -.040 | -.047 | -.031 |
| All-Weather Gear | Children or Educators vs. Neither |  |  | .039 | .021 |
|  | Children and Educators vs. Neither |  |  | .061 | .047 |
| Child Autonomy | Medium vs. Low |  |  | .008 | .029 |
|  | High vs. Low |  |  | -.008 | .034 |
| Affordances | Loose Parts |  |  | **.109*** | **.223***** |
|  | Gardening Elements |  |  | .021 | .015 |
|  | Natural Elements |  |  | .062 | .078 |
|  | Fixed Equipment |  |  | -.045 | -.033 |
|  | Portable Equipment |  |  | .069 | .067 |
| Interactions | Director Only OP Training x Loose Parts |  |  |  | -.053 |
|  | Educator Only OP Training x Loose Parts |  |  |  | .027 |
|  | Both with OP Training x Loose Parts |  |  |  | **-.186***** |
|  | Director Only OP Training x Fixed Equipment |  |  |  | .033 |
|  | Educator Only OP Training x Fixed Equipment |  |  |  | -.020 |
|  | Both with OP Training x Fixed Equipment |  |  |  | -.051 |
|  | Director Only OP Training x Autonomy |  |  |  | -.016 |
|  | Educator Only OP Training x Autonomy |  |  |  | **.110**** |
|  | Both with OP Training x Autonomy |  |  |  | .014 |
|  |  |  |  |  |  |
|  | Adjusted R^2^ and Significance of R^2^ Change | .011* | .036*** | .067*** | .097*** |
|  | N (weighted) | 920 | 920 | 920 | 920 |

* *p*<.05, ** *p*<.01, *** *p*<.001

Table A6: Hierarchical Multiple Regression Models for Winter Frequency, Preschool-Age Programs

|  |  | Winter Frequency in PS Centres: Betas & Significance | | | |
| --- | --- | --- | --- | --- | --- |
|  |  | Only Centre Characteristics | Add Staff Characteristics | Add Physical Environment and Additional Characteristics | Add Interactions |
| Auspice | Non-profit vs. For-profit | .035 | .027 | -.006 | .002 |
| Rural/Urban | Rural vs. Urban Postal Code | -.027 | -.023 | -.031 | -.029 |
| Centre Ages | Both Ages vs. Just I/T | **-.618***** | **-.620***** | **-.612***** | **-.612***** |
| OP Area Size | Smaller/Off-Site vs. As required | .031 | .023 | -.010 | -.002 |
|  | Larger vs. As required | -.018 | -.017 | -.027 | -.031 |
|  | Much larger vs. As required | .039 | .038 | .016 | .017 |
| Access to OP Area | Direct vs. Not | .018 | .011 | -.017 | -.015 |
| OP Training | Director Only vs. None |  | .024 | .011 | .006 |
|  | Educator Only vs. None |  | -.016 | -.021 | -.008 |
|  | Director and Educators vs. None |  | **.080**** | .032 | .038 |
| Staff Tenure | Medium vs. Low |  | .004 | -.022 | -.020 |
|  | High vs. Low |  | -.014 | -.023 | -.023 |
| All-Weather Gear | Children or Educators vs. Neither |  |  | -.004 | .004 |
|  | Children and Educators vs. Neither |  |  | -.009 | -.009 |
| Child Autonomy | Medium vs. Low |  |  | .043 | **.075**** |
|  | High vs. Low |  |  | **.108***** | **.155***** |
| Affordances | Loose Parts |  |  | **.181***** | **.129**** |
|  | Gardening Elements |  |  | -.033 | -.022 |
|  | Natural Elements |  |  | **.128***** | **.130***** |
|  | Fixed Equipment |  |  | -.030 | -.076 |
|  | Portable Equipment |  |  | -.022 | -.018 |
| Interactions | Director Only OP Training x Loose Parts |  |  |  | -.005 |
|  | Educator Only OP Training x Loose Parts |  |  |  | **.082**** |
|  | Both with OP Training x Loose Parts |  |  |  | .045 |
|  | Director Only OP Training x Fixed Equipment |  |  |  | **.084**** |
|  | Educator Only OP Training x Fixed Equipment |  |  |  | .034 |
|  | Both with OP Training x Fixed Equipment |  |  |  | -.007 |
|  | Director Only OP Training x Autonomy |  |  |  | .017 |
|  | Educator Only OP Training x Autonomy |  |  |  | .010 |
|  | Both with OP Training x Autonomy |  |  |  | **.070*** |
|  |  |  |  |  |  |
|  | Adjusted R^2^ and Significance of R^2^ Change | .393*** | .397* | .451*** | .462*** |
|  | N (weighted) | 1,033 | 1,033 | 1,033 | 1,033 |

* *p*<.05, ** *p*<.01, *** *p*<.001

Table A7: Hierarchical Multiple Regression Models for Risky Play, Infant/Toddler Programs

|  |  | Risky Play in I/T Centres: Betas & Significance | | | |
| --- | --- | --- | --- | --- | --- |
|  |  | Only Centre Characteristics | Add Staff Characteristics | Add Physical Environment and Additional Characteristics | Add Interactions |
| Auspice | Non-profit vs. For-profit | .024 | .009 | -.056 | -.061 |
| Rural/Urban | Rural vs. Urban Postal Code | .030 | .036 | .011 | .007 |
| Centre Ages | Both Ages vs. Just I/T | -.048 | **-.065*** | -.042 | -.052 |
| OP Area Size | Smaller/Off-Site vs. As required | **.160***** | **.139**** | .061 | .058 |
|  | Larger vs. As required | -.057 | -.068 | -.060 | -.056 |
|  | Much larger vs. As required | .097* | .094 | .054 | .053 |
| Access to OP Area | Direct vs. Not | **.086**** | **.087**** | **.056** | **.061*** |
| OP Training | Director Only vs. None |  | .024 | .011 | .014 |
|  | Educator Only vs. None |  | .016 | .005 | .014 |
|  | Director and Educators vs. None |  | **.166***** | **.098**** | **.087**** |
| Staff Tenure | Medium vs. Low |  | -.031 | -.047 | -.043 |
|  | High vs. Low |  | **-.087*** | **-.096**** | **-.084*** |
| All-Weather Gear | Children or Educators vs. Neither |  |  | .043 | .058 |
|  | Children and Educators vs. Neither |  |  | **.086**** | **.087**** |
| Child Autonomy | Medium vs. Low |  |  | **.165***** | **.213***** |
|  | High vs. Low |  |  | **.230***** | **.296***** |
| Affordances | Loose Parts |  |  | **.164***** | .096 |
|  | Gardening Elements |  |  | **.119**** | **.125**** |
|  | Natural Elements |  |  | .008 | .005 |
|  | Fixed Equipment |  |  | **.138***** | **.115*** |
|  | Portable Equipment |  |  | -.030 | -.043 |
| Interactions | Director Only OP Training x Loose Parts |  |  |  | .014 |
|  | Educator Only OP Training x Loose Parts |  |  |  | **.101**** |
|  | Both with OP Training x Loose Parts |  |  |  | .069 |
|  | Director Only OP Training x Fixed Equipment |  |  |  | .002 |
|  | Educator Only OP Training x Fixed Equipment |  |  |  | -.012 |
|  | Both with OP Training x Fixed Equipment |  |  |  | .051 |
|  | Director Only OP Training x Autonomy |  |  |  | .043 |
|  | Educator Only OP Training x Autonomy |  |  |  | **.102**** |
|  | Both with OP Training x Autonomy |  |  |  | .035 |
|  |  |  |  |  |  |
|  | Adjusted R^2^ and Significance of R^2^ Change | .046*** | .067*** | .254*** | .265** |
|  | N (weighted) | 921 | 921 | 921 | 921 |

* *p*<.05, ** *p*<.01, *** *p*<.001

Table A8: Hierarchical Multiple Regression Models for Risky Play, Preschool-Age Programs

|  |  | Risky Play in PS Centres: Betas & Significance | | | |
| --- | --- | --- | --- | --- | --- |
|  |  | Only Centre Characteristics | Add Staff Characteristics | Add Physical Environment and Additional Characteristics | Add Interactions |
| Auspice | Non-profit vs. For-profit | **.116***** | **.082**** | .010 | .09 |
| Rural/Urban | Rural vs. Urban Postal Code | **.087**** | **.097**** | **.058*** | **.065*** |
| Centre Ages | Both Ages vs. Just I/T | **.085**** | **.071*** | **.082**** | **.078**** |
| OP Area Size | Smaller/Off-Site vs. As required | **.194***** | **.178***** | **.093*** | **.1*** |
|  | Larger vs. As required | .043 | .043 | .018 | .02 |
|  | Much larger vs. As required | **.138**** | **.130**** | .064 | .063 |
| Access to OP Area | Direct vs. Not | **.090**** | **.071*** | -.002 | .013 |
| OP Training | Director Only vs. None |  | .060 | .017 | .02 |
|  | Educator Only vs. None |  | .056 | .045 | .053 |
|  | Director and Educators vs. None |  | **.204***** | **.101**** | **.092**** |
| Staff Tenure | Medium vs. Low |  | -.030 | **-.075*** | **-.066*** |
|  | High vs. Low |  | -.010 | -.035 | -.044 |
| All-Weather Gear | Children or Educators vs. Neither |  |  | **.065*** | **.073*** |
|  | Children and Educators vs. Neither |  |  | .0049 | .051 |
| Child Autonomy | Medium vs. Low |  |  | **.095**** | **.094**** |
|  | High vs. Low |  |  | **.242***** | **.237***** |
| Affordances | Loose Parts |  |  | **.249***** | **.207***** |
|  | Gardening Elements |  |  | .067 | .066 |
|  | Natural Elements |  |  | .074 | .07 |
|  | Fixed Equipment |  |  | **.146***** | **.107*** |
|  | Portable Equipment |  |  | -.065 | -.054 |
| Interactions | Director Only OP Training x Loose Parts |  |  |  | .007 |
|  | Educator Only OP Training x Loose Parts |  |  |  | -.005 |
|  | Both with OP Training x Loose Parts |  |  |  | .06 |
|  | Director Only OP Training x Fixed Equipment |  |  |  | **.066*** |
|  | Educator Only OP Training x Fixed Equipment |  |  |  | .005 |
|  | Both with OP Training x Fixed Equipment |  |  |  | .03 |
|  | Director Only OP Training x Autonomy |  |  |  | **.074*** |
|  | Educator Only OP Training x Autonomy |  |  |  | -.046 |
|  | Both with OP Training x Autonomy |  |  |  | -.023 |
|  |  |  |  |  |  |
|  | Adjusted R^2^ and Significance of R^2^ Change | .053*** | .081*** | .282*** | .288* |
|  | N (weighted) | 1,040 | 1,040 | 1,040 | 1,040 |

* *p*<.05, ** *p*<.01, *** *p*<.001
