# Supplementary material for: Examining outdoor play associations in Canadian early learning and child care centres: Cross-sectional insights from the Measuring Early Childhood Outside survey
Source: PLoS One. 2026 Feb 11;21(2):e0331166. doi: 10.1371/journal.pone.0331166 (PMC12893580; doi:10.1371/journal.pone.0331166)
Supplement: Attachment — Submitted filename: PLOS ONE Response to Reviewers.docx [file pone.0331166.s005.docx]

Dear Editor and Reviewers,

Thank you for your thoughtful and helpful comments regarding our recent submission, *‘Examining outdoor play associations in Canadian early learning and child care centres: Cross-sectional insights from the Measuring Early Childhood Outside survey,* for consideration to *PLOS ONE*. We appreciate the encouraging feedback and acknowledgement of this paper as a worthwhile contribution to the literature. Additionally, the feedback provided was critical to strengthen the work within our revised manuscript. Please see below for our responses to the received comments from reviewers and the changes made in our revised manuscript.

| **Editor comment** | **Author Response** |
| --- | --- |
| 1. Please ensure that your manuscript meets PLOS ONE's style requirements, including those for file naming. | We have revised headings and text format, including Fig 1, to align with style templates. |
| 2. Please provide a complete Data Availability Statement in the submission form, ensuring you include all necessary access information or a reason for why you are unable to make your data freely accessible. If your research concerns only data provided within your submission, please write "All data are in the manuscript and/or supporting information files" as your Data Availability Statement. | We’ve updated the data availability statement in the submission form, including the link to access the dataset. All dataset files are available from the UBC Research Data Collection database.  <https://doi.org/10.5683/SP3/WSZ1W5>, |
| 3. When completing the data availability statement of the submission form, you indicated that you will make your data available on acceptance. We strongly recommend all authors decide on a data sharing plan before acceptance, as the process can be lengthy and hold up publication timelines. Please note that, though access restrictions are acceptable now, your entire data will need to be made freely accessible if your manuscript is accepted for publication. This policy applies to all data except where public deposition would breach compliance with the protocol approved by your research ethics board. If you are unable to adhere to our open data policy, please kindly revise your statement to explain your reasoning and we will seek the editor's input on an exemption. Please be assured that, once you have provided your new statement, the assessment of your exemption will not hold up the peer review process. | Our data have been deposited and are now accessible from the UBC Research Data Collection database.  <https://doi.org/10.5683/SP3/WSZ1W5>, |
| 4. Please include captions for your Supporting Information files at the end of your manuscript, and update any in-text citations to match accordingly. Please see our Supporting Information guidelines for more information. | We have added captions for all supporting information files at the end of our manuscript. |
| 5. If the reviewer comments include a recommendation to cite specific previously published works, please review and evaluate these publications to determine whether they are relevant and should be cited. There is no requirement to cite these works unless the editor has indicated otherwise. | No action required. |

| **Reviewer #1 comments** | **Author Response** |
| --- | --- |
| 1. The low and uneven provincial response rate (9.8%), exclusion of certain jurisdictions, and reliance on weighting raise concerns about representativeness. These issues should be discussed more explicitly as potential sources of bias, and the findings should be interpreted as associative rather than generalizable to all Canadian ELCC settings. | We have further emphasized the reduced generalizability of findings within the ‘Survey response rates and weighting’ subheading. The current ‘Limitations’ subheading outlines these limitations already, however, we added an additional sentence to explain the limited generalizability due to a low overall sample. |
| 2. The use of hierarchical multiple regression is appropriate for examining associations among the predictors, and the inclusion of interaction terms (training × affordances/autonomy) adds value. However, several analytic details need clarification: a. Describe how missing data were handled and report the sample size used for each model after exclusions. | We’ve reported how missing data were handled in the Methods – Analytical plan. In addition, we’ve included weighted sample sizes in each data table reporting model results. |
| 2. b. Provide checks for multicollinearity and model diagnostics (e.g., residual analysis, VIFs). | To check for multicollinearity, VIF was considered. We’ve included this in the Methods – Analytical plan section. |
| 2. c. Justify treating ordinal risky-play variables as continuous and consider presenting sensitivity or robustness checks. | We’ve included further information on the reliability of the new risky play outcome scale we derived for this study in the Methods – Outcome section |
| 2. d. Including marginal-effect or predicted-value plots for key interactions would improve interpretability. | We appreciate this feedback to support a more detailed explanation of the interaction terms. The interaction terms were selected within the literature and by what is known about the field of outdoor play and child care to-date. In lieu of plots, we’ve added more information on the selection of these interaction terms and the existing evidence that supports these terms in Methods – Analytic plan. |
| 3. Tables are comprehensive and informative. Minor editorial improvements could enhance readability: a. Ensure consistent terminology throughout (e.g., “off-site” vs. “larger-than-required” outdoor play areas).   b. Review for minor formatting or spacing inconsistencies. | We have reviewed all tables for terminology and formatting inconsistencies and made edits accordingly. |
| 3. Consider condensing portions of the Introduction that repeat background literature to improve flow and focus. | We have condensed some areas of the Introduction; however, we have also expanded on the framework of time, space and freedom as per Reviewer #2’s feedback. |

| **Reviewer #2 comments** | **Author Response** |
| --- | --- |
| 1. Clarification of the "Time, Space, and Freedom" Framework: While the "time, space, and freedom" framework is conceptually strong, its discussion in the manuscript is somewhat abstract. The authors are encouraged to provide concrete examples or case studies to illustrate how these factors influence the implementation of OP in real-world settings. Additionally, linking this framework more explicitly to the findings of the MECO survey would enhance its practical relevance. | We’ve expanded on the time, space and freedom framework in the Introduction section to include academic examples. We have also further related the findings back to this framework in the Discussion section. |
| 2. Detailed Description of MECO Survey Methods: The manuscript could benefit from a more detailed explanation of the MECO survey's data collection and analysis methods. Specifically, the authors should clarify the sampling strategy, the representativeness of the sample, and the measures taken to ensure the reliability and validity of the data. This would strengthen the transparency and credibility of the study. | The sampling strategy is currently outlined in the subheading ‘Survey distribution and promotion’ within the Materials and methods section. However, we have added a new sentence to clearly outline the strategy type (convenience sample).  The representativeness of the sample is provided in Table 1 and outlined in the ‘Survey response rates and weighting’ in the Materials and methods section. Limitations of the representativeness of the sample are further outlined in the Limitations section.  Reliability was sought via pilot testing (see ‘Survey development and pilot testing’ section).  Validity of the dataset was sought by comparing the sample to the target population (Table 1). Target population information related to auspice or detailed geographic region (urban/rural) are not available, and therefore a validity check on these variables could not be conducted. |
