# Supplementary material for: Examining outdoor play associations in Canadian early learning and child care centres: Cross-sectional insights from the Measuring Early Childhood Outside survey
Source: PLoS One. 2026 Feb 11;21(2):e0331166. doi: 10.1371/journal.pone.0331166 (PMC12893580; doi:10.1371/journal.pone.0331166)
Supplement: Attachment — Submitted filename: PLOS_ONE_Response_to_Reviewers_auresp_2.docx [file pone.0331166.s006.docx]

Dear Editor and Reviewers,

Thank you for your thoughtful and helpful comments regarding our revised submission for our manuscript, *‘Examining outdoor play associations in Canadian early learning and child care centres: Cross-sectional insights from the Measuring Early Childhood Outside survey,* for consideration to *PLOS ONE*. Please see below for our responses to the updated comments from reviewers and the changes made in our revised manuscript.

| **Editor comment** | **Author Response** |
| --- | --- |
| 1. References should not be cited in the conclusion section. | We have removed references in the conclusion section. |

| **Reviewer #2 comments** | **Author Response** |
| --- | --- |
| 1. The manuscript still requires some minor revisions. First, the overall length of the paper should be reduced. In particular, the Discussion and Limitations sections are overly lengthy and would benefit from more concise and focused discussion that directly reflects the key findings of the study. | We have condensed the Discussion and Limitations sections accordingly. |
| 2. Second, the Conclusions section is too verbose and should be further refined. The conclusions should clearly and succinctly summarize the main findings, practical implications, and directions for future research. In addition, references should not be cited in the Conclusions section. | We have condensed the conclusion. We have also removed references in the conclusion section. |
